# Scrotal Cellulitis in the Setting of IL-17A Inhibitor Therapy: A Case Report

**DOI:** 10.7759/cureus.91693

**Published:** 2025-09-05

**Authors:** Jamie McDermott, Ayman Anasi, Nima Sadeghi, Vance Bowen, Avtar Singh

**Affiliations:** 1 Medicine, Midwestern University Arizona College of Osteopathic Medicine, Glendale, USA; 2 Internal Medicine, Carl T Hayden VA Medical Center, Phoenix, USA; 3 Medicine, University of Arizona College of Medicine, Phoenix, USA

**Keywords:** cellulitis, ixekizumab, psoriasis, psoriatic arthritis, scrotal cellulitis, scrotum, thrombophlebitis

## Abstract

Ixekizumab, an IL-17A inhibitor, is commonly used to treat moderate-to-severe plaque psoriasis and psoriatic arthritis, with a well-documented risk of mucocutaneous *Candida* infections, though its role in bacterial infections is less defined. We present the case of a 62-year-old male on ixekizumab who developed scrotal cellulitis with subsequent septic thrombophlebitis, despite no clear entry point. His condition initially improved with broad-spectrum antibiotics, but later worsened, requiring further imaging and multidisciplinary management. The IL-17 plays a key role in neutrophil recruitment and skin barrier integrity, and its inhibition may impair bacterial defense, potentially increasing the risk of severe infections. This case underscores the need for clinicians to recognize bacterial infections as a possible complication of IL-17 blockade and highlights the importance of early intervention and close monitoring in immunomodulatory patients.

## Introduction

Scrotal cellulitis is a bacterial skin infection affecting the scrotum, often extending to the perineum and nearby tissues [1]. It is marked by redness, swelling, warmth, and tenderness, and may be accompanied by systemic symptoms such as fever and malaise. The condition develops when bacteria, most often beta-hemolytic* Streptococcus* or *Staphylococcus aureus*, invade the skin and subcutaneous layers [1]. Contributing factors include poor hygiene, diabetes, obesity, immune suppression, and disruption of the skin barrier from causes such as fungal infections, dermatitis, or recent trauma [2]. Individuals receiving immunomodulatory therapy, including biologics for autoimmune diseases, may be more prone to infection due to altered immune responses. Psoriasis is a chronic autoimmune disease characterized by rapid skin cell turnover, resulting in inflammation and scaling [2]. Plaque psoriasis, the most common form, typically presents as red, scaly plaques on the scalp, elbows, and knees, and can be managed with topical agents, phototherapy, systemic immunosuppressants, or biologics that target IL-17 or IL-23 pathways [3].

Ixekizumab, an IL-17A antagonist, is a biologic therapy approved for moderate to severe plaque psoriasis and psoriatic arthritis [4,5]. It plays a key role in antifungal immunity and skin barrier defense, and its inhibition can increase susceptibility to infections. Ixekizumab is most often associated with mucocutaneous infections, particularly *Candida*, but its risk for bacterial infections such as cellulitis is less well defined compared to TNF-α inhibitors [6]. This case report describes an unusual instance of scrotal cellulitis in a patient with plaque psoriasis receiving ixekizumab, highlighting a potential but underrecognized bacterial risk. Given IL-17’s involvement in neutrophil recruitment, this case underscores the importance of careful monitoring and further research into infectious complications associated with IL-17 inhibition.

## Case presentation

We present the case of a 62-year-old male with a past medical history significant for psoriatic arthritis on ixekizumab therapy, who presented to the emergency department with scrotal and right groin swelling and erythema. The patient reported a fever of 100.3°F, chills, nausea, and vomiting on the morning of presentation. The day prior, he had noticed swelling, erythema, and tenderness beginning in the scrotum and migrating to the right groin and inguinal region. His pain was initially rated 7/10 in severity, increasing to 10/10 without medication. Two weeks earlier, he had completed a 10-day course of amoxicillin for an ear infection.

**Figure 1:**
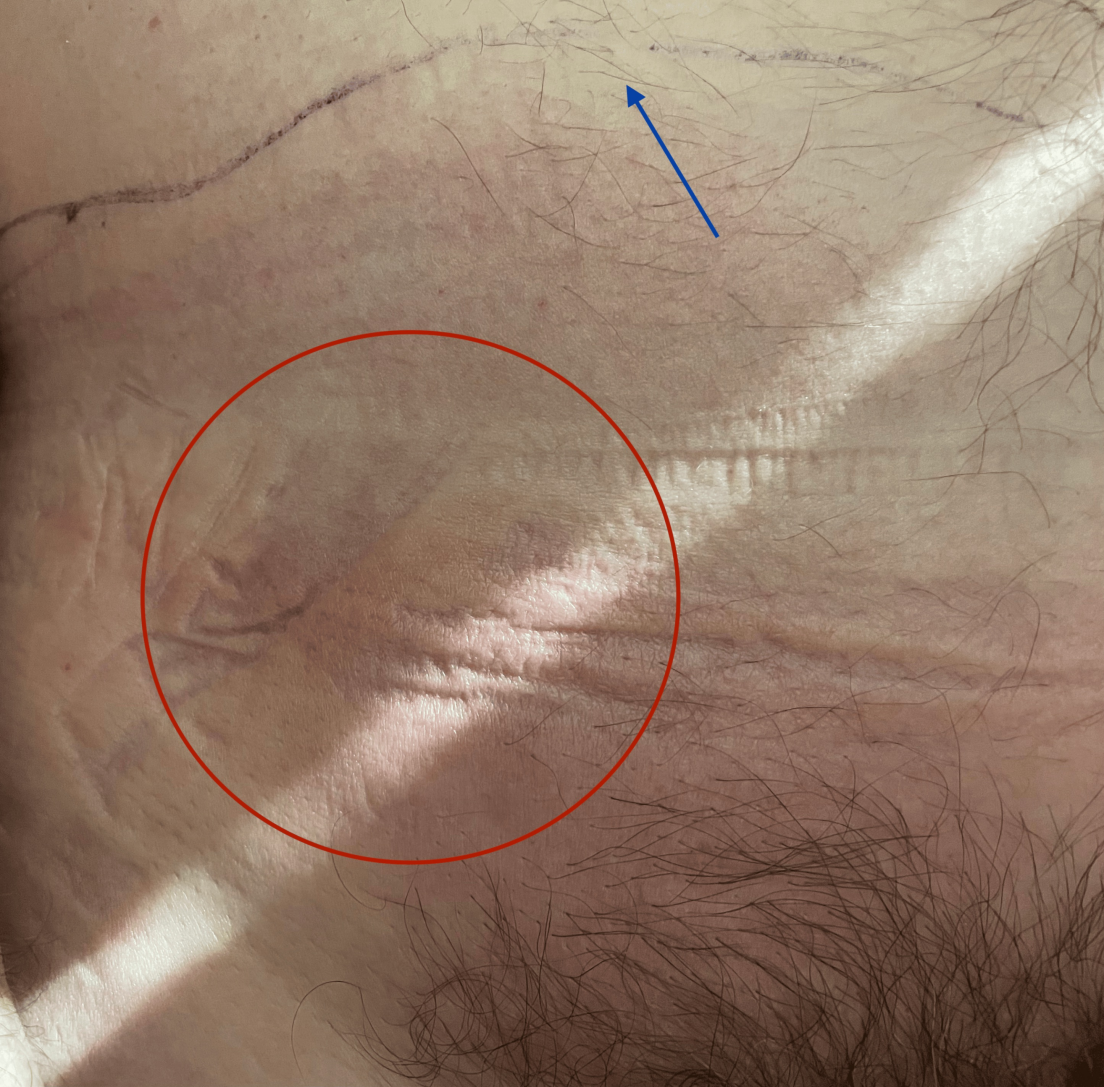
Localized erythema with marked margins in suspected soft tissue infection Localized erythema (red circle) surrounding an area of concern for soft tissue infection. A faintly demarcated line (blue arrow) represents the margin marked by the clinical team to monitor progression of erythema and potential spread of infection.

In the emergency department, he received intravenous (IV) antibiotics (clindamycin 600 mg, vancomycin 1000 mg, and piperacillin-tazobactam 3.375 g) and IV fluids (potassium chloride at 10 mEq/hour and 0.9% sodium chloride, 1 L). Borders were drawn with a surgical marker to monitor the spread of erythema (Figure 1). Urology was consulted, and blood cultures grew *Streptococcus pyogenes*. The patient was admitted to the internal medicine service with a diagnosis of cellulitis. On physical exam, there was no visible entry point to isolate the initial site of bacterial invasion.

Repeat cultures on admission day three showed no growth, and the patient was started on ceftriaxone and linezolid per recommendations from Infectious Disease. General surgery was consulted after worsening cellulitis and erythema began to spread to the flank region. Linezolid was discontinued on day five due to a drug eruption on the patient’s backside. A repeat CT of the abdomen and pelvis was performed the following day and reportedly showed improving cellulitis, minimal fat stranding, and no evidence of fascial air (the imaging is currently unavailable for review).

**Figure 2:**
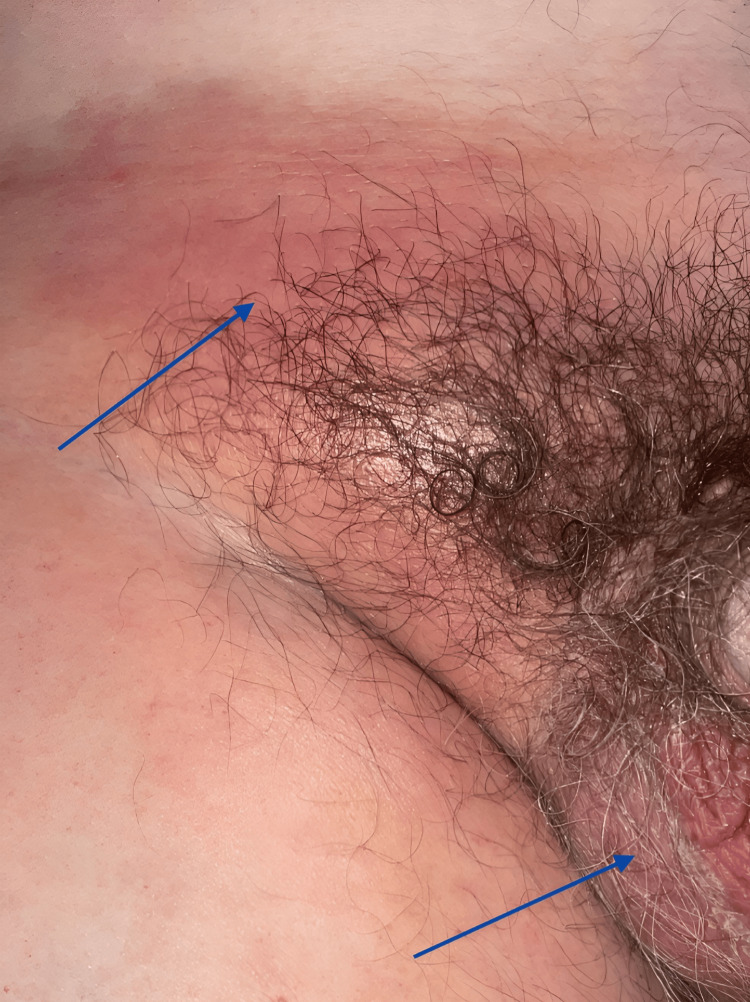
Progression of right inguinal erythema and swelling with improvement in scrotal edema Follow-up clinical image obtained one week after initial presentation, demonstrating increased erythema and swelling localized to the right inguinal region (top blue arrow). The patient reported worsening pain in the area (bottom blue arrow) despite a noticeable reduction in scrotal swelling.

One week after admission, the patient reported significant improvement in scrotal pain and swelling; however, he experienced worsening discomfort in the right inguinal region (Figure 2). The medical team considered a vascular ultrasound to evaluate for possible abscess or thrombophlebitis. Ultimately, a repeat CT of the abdomen and pelvis was obtained and interpreted as showing signs of septic thrombophlebitis.

The patient responded well to intravenous ceftriaxone 2 g once daily and therapeutic anticoagulation with enoxaparin 1 mg/kg every 12 hours, along with supportive care for the drug eruption. Symptoms gradually improved, and he was transitioned to oral amoxicillin-clavulanate 875 mg/125 mg twice daily to complete a four-week course. At follow-up, he remained free of recurrent infection. 

## Discussion

Cutaneous side effects from biologic therapies are well recognized, but this case stands out for involving a severe bacterial skin infection in a patient treated with ixekizumab, an IL-17A inhibitor. Several factors complicate interpretation, including recent antibiotic use before symptom onset, background inflammation from psoriatic arthritis, and the lack of a clear source of bacterial entry. These considerations suggest that multiple mechanisms may have contributed to the infection rather than IL-17 inhibition alone.

The IL-17 is a pro-inflammatory cytokine produced by Th17 cells, γδ T cells, and innate lymphoid cells and is central to mucocutaneous host defense. Its downstream effects include upregulation of chemokines such as CXCL1 and CXCL8, which recruit neutrophils to sites of infection, and stimulation of epithelial cells to produce antimicrobial peptides [7-9]. These mechanisms provide a rapid 'first-line' barrier against extracellular bacteria and fungi. Inhibition of IL-17 therefore has a dual impact: (1) it reduces neutrophil trafficking, impairing bacterial clearance, and (2) it compromises epithelial barrier function, potentially facilitating deeper microbial invasion [9].

The most consistently reported infections associated with IL-17 inhibitors are mucocutaneous *Candida*, reflecting IL-17’s essential role in antifungal immunity [7,8]. Emerging evidence, beyond the well-characterized *Candida* risk, suggests that IL-17A blockade may predispose patients to serious bacterial infections. A meta-analysis of randomized and extension trials estimated overall infection exposure-adjusted incidence rates (EAIRs) of ~57.8 and serious infections of ~1.1 per 100 patient-years with IL-17 inhibitors, with *Candida* notably more common but serious infections still rare [10]. Clinical trials of ixekizumab and secukinumab have reported mild to moderate bacterial infections, particularly upper respiratory tract infections, but serious infections have been documented in post-marketing surveillance and case reports [11,12]. For example, cases of recurrent *S. aureus *skin infections, disseminated candidiasis, and cellulitis requiring hospitalization have been described in patients receiving IL-17 blockade [13,14].

The occurrence of scrotal cellulitis progressing to septic thrombophlebitis in our patient, without an identifiable portal of entry, raises the question of whether IL-17 inhibition might predispose to bacterial dissemination in otherwise uncommon anatomic sites. It is plausible that diminished neutrophil recruitment under IL-17 blockade permitted local infection to progress unchecked, ultimately leading to vascular involvement. The patient’s recent antibiotic exposure may also have altered the microbiome and immune readiness, compounding this susceptibility. Antibiotic-induced dysbiosis is increasingly recognized as a contributor to impaired host defense, particularly in immunomodulated patients.

Management of this case was further complicated by a drug eruption, limiting antibiotic options and underscoring the need for multidisciplinary care. Ultimately, the patient responded to ceftriaxone and anticoagulation, but his course highlights both the potential severity of infection in patients on IL-17 inhibitors and the diagnostic uncertainty they present. While IL-17 blockade remains an effective therapy for psoriasis and psoriatic arthritis, clinicians should maintain vigilance for bacterial as well as fungal infections, particularly those with atypical presentations or rapid progression. Further accumulation of case reports and registry data will be essential to better define the infectious risk profile of IL-17 inhibitors and to develop evidence-based monitoring strategies.

## Conclusions

This case suggests the possibility of a severe presentation of scrotal cellulitis progressing to septic thrombophlebitis in a patient receiving ixekizumab for psoriatic arthritis. While IL-17 inhibitors are primarily associated with an increased risk of mucocutaneous *Candida* infections, this report raises concerns about their potential impact on bacterial immunity, particularly in skin and soft tissue infections. The absence of a clear entry point and the patient’s recent antibiotic exposure further complicate the understanding of his susceptibility. Given the widespread use of IL-17 blockade in autoimmune diseases, clinicians should maintain a high index of suspicion for bacterial infections in these patients, especially when presenting with rapidly progressing cellulitis. Multidisciplinary collaboration is essential for timely diagnosis and management, and further research is needed to better characterize the infectious risks associated with IL-17 inhibition.
